# Neutrophil and Colorectal Cancer

**DOI:** 10.3390/ijms26010006

**Published:** 2024-12-24

**Authors:** Hideyuki Masui, Kenji Kawada, Kazutaka Obama

**Affiliations:** 1 Department of Surgery, Graduate School of Medicine, Kyoto University, Kyoto 606-8507, Japan; hmasui@kuhp.kyoto-u.ac.jp (H.M.); kobama@kuhp.kyoto-u.ac.jp (K.O.); 2 Department of Surgery, Hirakata Kohsai Hospital, Osaka 573-0153, Japan; 3 Department of Surgery, Kurashiki Central Hospital, Okayama 710-8602, Japan

**Keywords:** cancer, tumor microenvironment, tumor-associated neutrophil, metastasis, chemokine

## Abstract

Colorectal cancer (CRC) is often associated with metastasis and recurrence and is the leading cause of cancer-related mortality. In the progression of CRC, recent studies have highlighted the critical role of neutrophils, particularly tumor-associated neutrophils (TANs). TANs have both tumor-promoting and tumor-suppressing activities, contributing to metastasis, immunosuppression, angiogenesis, and epithelial-to-mesenchymal transition. Tumor-promoting TANs promote tumor growth by releasing proteases, reactive oxygen species, and cytokines, whereas tumor-suppressing TANs enhance immune responses by activating T cells and natural killer cells. Understanding the mechanisms underlying TAN mobilization, plasticity, and their role in the tumor microenvironment has revealed potential therapeutic targets. This review provides a comprehensive overview of TAN biology in CRC and discusses both the tumor-promoting and tumor-suppressing functions of neutrophils. Novel therapeutic approaches targeting TANs, such as chemokine receptor antagonists, aim to modulate neutrophil reprogramming and offer promising avenues for improving treatment outcomes of CRC.

## 1. Introduction

Colorectal cancer (CRC) is the third most common malignancy worldwide, following breast and lung cancer, and is the second leading cause of cancer-related death [1]. Due to the spread of western diets and lifestyle changes, the number of deaths caused by CRC has increased by over 30% in the past 15 years and is projected to rise by 25% in the next decade [2,3]. Despite improvements in treatment and screening, the mortality rate of CRC remains still high, usually due to distant metastasis or postoperative recurrence. The treatment of CRC primarily includes surgery, chemotherapy, chemoradiotherapy, and molecular-targeted therapy, with strategies tailored to patients at various stages. Molecular-targeted drugs, such as vascular endothelial growth factor (VEGF), epidermal growth factor receptor (EGFR), human epidermal growth factor receptor 2 (HER2), and v-RAF murine sarcoma viral oncogene homolog B (BRAF), have significantly improved patient survival [4]. Furthermore, immune checkpoint inhibitors targeting programmed cell death protein 1 (PD-1), programmed cell death ligand 1 (PD-L1), and cytotoxic T lymphocyte-associated antigen-4 (CTLA-4) have shown promising results in activating T cells to exert antitumor effects [5]. However, immune checkpoint inhibitors are effective in less than 10% of patients with microsatellite instability-high (MSI-H) or mismatch repair-deficient (dMMR) CRC, where the tumor microenvironment (TME) is characterized by high T cell infiltration and elevated immune checkpoint expression [6]. In contrast, immune checkpoint inhibitors are ineffective in most patients with microsatellite stable (MSS) and mismatch repair-proficient (pMMR) CRC [7].

**Figure 1:**
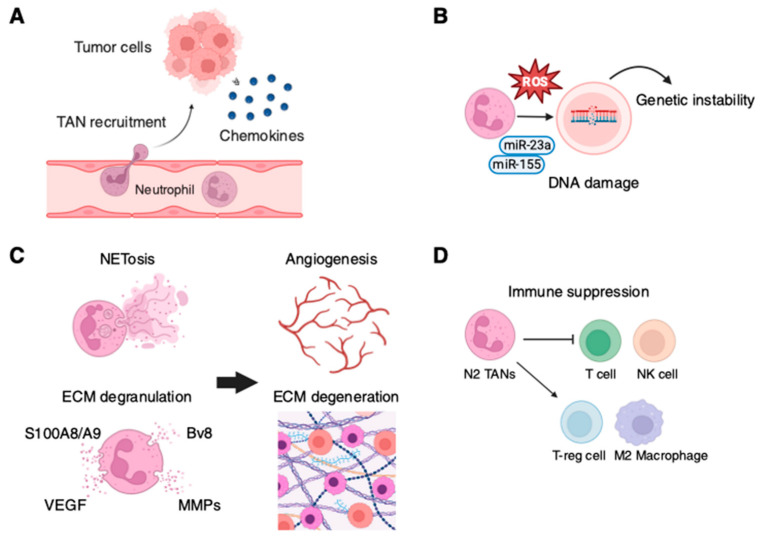
Multiple functions of tumor-associated neutrophils (TANs). (**A**) Recruitment of TANs by tumor-derived chemokines. (**B**) Induction of genetic instability through ROS production and microRNAs (e.g., miR-23a, miR-155), leading to DNA damage. (**C**) Promotion of extracellular matrix (ECM) remodeling via degranulation and degradation involving MMPs, Bv8, and S100A8/A9, facilitating angiogenesis driven by VEGF. (**D**) Immunosuppression mediated by N2 TANs through interactions with T cells, NK cells, and T-reg cells, and polarization of macrophages into the M2 phenotype. Created with BioRender.com.

In line with this trend, recent studies have increasingly recognized the crucial role of the immune system in cancer progression and treatment outcomes, highlighting the importance of the TME. Among various immune cells, neutrophils have gained attention for their dual roles in cancer [8]. While traditionally known for their functions in fighting infections, emerging evidence suggests that neutrophils influence tumor growth, metastasis, and response to therapy. This has led to a growing interest in understanding the complex interactions between neutrophils and cancer cells, particularly in the context of CRC. Understanding these interactions could unveil novel therapeutic targets and strategies to improve therapeutic efficacy and patient prognosis. In this review, we provide a comprehensive overview of the multifaceted roles of neutrophils in CRC (Figure 1).

## 2. Multifaceted Roles of Neutrophils in Cancer

Neutrophils are the first responders to infection and inflammation and are characterized by their short lifespan and inability to proliferate. They are pivotal components of the innate immune system, constituting approximately 70% of all peripheral leukocytes in humans and 10−20% in mice [9]. In comparison, the adaptive immune system relies on T cells and B cells to provide antigen-specific responses and immunological memory. Neutrophils have a wide range of functions in fighting pathogens, including phagocytosis, generation of reactive oxygen species (ROS), degranulation with the release of proteases and cytotoxic granules, recruitment of other immune cells, and the formation of neutrophil extracellular traps (NETs) [10]. Although the functional importance of neutrophils in cancer research has been underestimated, recent studies have highlighted the importance of neutrophils in cancer pathogenesis, especially in CRC [5,11,12]. Advances in technologies such as in vivo imaging, high-dimensional transcriptome analysis, single-cell analysis, and epigenomic analysis have prompted the re-evaluation of neutrophil biology in cancer [13].

The innate immune system contributes to cancer initiation and progression through inflammation, a concept historically recognized as the Virchow hypothesis. Numerous studies have reported that peripheral blood neutrophil counts are elevated in patients with various types of cancers. High frequencies of circulating neutrophils and tumor-infiltrating neutrophils are strongly associated with poor prognosis in patients with CRC, as evidenced by the fact that the neutrophil-to-lymphocyte ratio is an independent prognostic indicator [14,15,16]. Neutrophils not only constitute a predominant population of circulating cells in the human bloodstream but also exhibit a remarkable tendency to infiltrate the intricate milieu of the TME in substantial quantities [17]. Notably, neutrophils are found to infiltrate solid tumors, including CRC [18,19,20], and intratumoral neutrophils have been reported to be the most adverse prognostic cell type among all tumor-infiltrating leukocyte populations based on a pan-cancer evaluation of over 3000 solid tumors comprising 14 different cancer types. The study utilized CIBERSORT, a computational approach, to infer leukocyte representation in bulk tumor transcriptomes [19]. The clinicopathological significance of neutrophil-rich colorectal carcinoma has recently been emphasized, with findings suggesting its impact on patient prognosis and tumor behavior [21]. Tumor-associated neutrophils (TANs) are recognized as a key component of the TME and are actively involved in various stages of cancer progression including CRC [5,10].

Nevertheless, in certain contexts, TANs have also been associated with a favorable prognosis in CRC [20,22,23], although only a limited number of studies have shown that TANs may inhibit CRC progression, mainly in the early stages of CRC. This will be discussed further in the following section. The relationship between tumor-infiltrating neutrophils and the prognosis remains a topic of debate, and the dual role of neutrophils in promoting and suppressing cancer remains undeniable.

## 3. Tumor-Associated Neutrophils (TANs)

The TME is composed of several host cells that suppress or promote cancer aggressiveness, including TANs, fibroblasts (cancer-associated fibroblasts: CAFs), macrophages (tumor-associated macrophages: TAMs), and mesenchymal stem cells [24]. Within the TME, neutrophils are highly dynamic cells that interact with adaptive immune cells such as T cells, B cells, natural killer cells, and tumor cells. Depending on the situation, neutrophils can exhibit antitumor functions through cytotoxic activity or tumor-promoting functions by facilitating immunosuppression, angiogenesis, cancer cell motility, epithelial-to-mesenchymal transition (EMT), and NET formation [10]. Therefore, identifying the biological events underlying their antitumor and tumor-promoting functions is crucial for realizing their therapeutic potential [25].

Historically, the understanding of immune cells in cancer has focused primarily on adaptive immune cells. The classification of immune cells has often followed the “Th1/Th2” paradigm, which has been extended to other immune cell populations. Among key players, macrophages received significant attention and were categorized into the “M1/M2” status, with an antitumorigenic phenotype and a pro-tumorigenic phenotype. Likewise, TANs also exhibit considerable plasticity and can be polarized into either an antitumorigenic “N1” phenotype or a pro-tumorigenic “N2” phenotype, while most studies on neutrophils in cancer have reported on the pro-tumorigenic role. As research on TANs has progressed, this hypothesis has been further validated, showing that neutrophils play dual roles that are closely associated with cancer prognosis [17] (Table 1).

N1 neutrophils exhibit increased cytotoxicity and decreased immunosuppressive capacity by producing tumor necrosis factor-α (TNF-α), intercellular adhesion molecule-1 (ICAM-1), ROS, and Fas, while decreasing arginase expression. In contrast, N2 neutrophils support tumor expansion by expressing arginase, matrix metalloproteinase-9 (MMP-9), VEGF, and chemokines such as CCL2, CCL5, and CXCL4 [5,24]. Transforming growth factor-β (TGF-β) signaling promotes the N2 phenotype, whereas the inhibition of TGF-β signaling induces the antitumoral N1 phenotype [27,37]. Interferon-β (IFN-β), interferon-γ (IFN-γ), and TNF-α have also been reported to induce the N1 phenotype [38,39].

Surface markers, transcriptional regulators, and cytokine profiles of neutrophils have not yet been investigated, while the functional plasticity of neutrophils is often related to their activation states rather than to a specific cellular phenotype [40]. Moreover, neutrophils are highly sensitive cells that respond rapidly and variably to host, tissue, and environmental factors [41,42]. It is also unclear whether intermediate N0 status exists [8]. Finally, it is worth noting that the heterogeneity of TANs extends far beyond the simple N1/2 classification, with more than nine different subsets already reported [43].

## 4. Antitumor Role of TANs

TANs exert their inhibitory effect on cancer tissues by directly killing cancer cells via the secretion of cytotoxic substances, such as ROS, nitric oxide (NO), and neutrophil elastase (NE). They also exert antitumor effects by expressing co-stimulatory receptors, including 4-1BBL, OX40L, and CD86, which activate T cells and induce secretion of IFN-γ [5,44]. H_2_O_2_ secreted by neutrophils depends on Ca^2+^ channels to induce cancer cell death by modulating the expression of transient receptor potential cation channel subfamily M member 2, thereby inhibiting cancer cell growth [45]. Signaling through TNF-α receptors on neutrophils stimulates NO production via hepatocyte growth factor and MET kinase pathways, resulting in the NO-mediated destruction of tumor cells [46].

N1 TANs also release a variety of chemokines and cytokines that stimulate the proliferation and activation of immune cells, such as T cells, NK cells, and dendritic cells (DCs), thus initiating antitumor immune responses [47]. Cytokines secreted by TANs include CCL3, CXCL10, TNF, and interleukin (IL)-12, which contribute to the recruitment and activation of CD8^+^ T cells [48]. Studies on human CRC have reported that the interactions between TANs and CD8^+^ T cells are associated with improved survival by enhancing T cell activation and proliferation [20]. In a murine CRC model, neutrophils lacking IL-1 receptor-associated kinase M (IRAK-M) exhibited reduced expression of PD-L1 and CD11b, whereas increased levels of CD40 and CD80 enhanced the antitumor immune response of T cells [49]. Furthermore, CD177^+^ TANs predicted better prognosis in CRC by suppressing epithelial cell tumorigenesis, as demonstrated in a colitis-associated cancer model [26]. In the early stages of cancer, TAN subsets from CD11b^+^CD15^high^CD10^−^CD16^low^ immature progenitor cells exhibit antitumor function, and CD62L^low^CD54^high^ neutrophils promote T cell proliferation and IFN-γ release, which activate the antitumor activity of neutrophils [44,50]. IFN signaling enhances the antitumor activity of neutrophils by upregulating the expression of TNF, ICAM-1, and CCL3, while downregulating arginase-1 levels [51]. TANs activated by IFN further boost the activity of NK cells secreting IL-18 and promote DC activation by releasing TNF [52]. Additionally, neutrophil-derived VEGF-A165b is involved in the inhibition of angiogenesis [53].

These findings have been validated in early-stage CRC models, where neutrophils inhibit IL-17−driven inflammation and cancer progression by controlling the bacterial load and diversity [54]. Neutrophil-specific IL-1 signaling inhibits IL-17−induced intestinal inflammation and cancer invasion in mice [54,55]. Similarly, blocking IL-1 receptor type 1 in neutrophils reduces the antibacterial capability, resulting in greater bacterial invasion of tumors and accelerated inflammation and cancer progression [55].

Although TANs exhibit antitumor effects as described above, these findings have not been as extensively studied as the tumor-promoting effects discussed in the next section.

## 5. Role of Neutrophils in Enhancing Tumor Progression

Neutrophils play a critical role in tumor progression. Inflammation is a key factor in cancer initiation through tissue damage and involves a specific subset of neutrophils that contributes to cancer pathogenesis. The relationship between inflammation and cancer has been validated in liver cancer, gastric cancer, and CRC [56,57]. Inflammatory bowel disease, particularly ulcerative colitis, is associated with an increased risk of developing CRC, characterized by inflammatory cell infiltration and elevated levels of inflammatory cytokines (e.g., IL-1β, IL-6, and TNFα) and chemokines (e.g., CCL2, and CXCL1) [58,59].

Neutrophils contribute to tumor development either by directly inducing genetic instability and promoting cell proliferation or by indirectly suppressing the antitumor immune response and promoting metastasis [13]. Neutrophils release a variety of factors, including NE, MMPs, ROS, VEGF, and prokineticin-2 (Bv8), all of which can influence tumor development.

The direct carcinogenic effects of neutrophils are related to ROS production, leading to tissue damage and genetic instability, which has been reported to increase mutational load in inflammation-driven mouse models of intestinal cancer [60]. Excessive ROS production by neutrophils is closely associated with NET formation (also known as NETosis), a process characterized by chromatin decondensation. During this process, the DNA repair protein PCNA, normally present in the cytoplasm, is translocated to the nucleus in response to nicotinamide adenine dinucleotide phosphate (NADPH)-dependent ROS production in humans [61]. Key components, such as Toll-like receptor-9, cyclic GMP-AMP synthase, and NOD-like receptor protein 3 and 2, which are absent in melanoma, have been identified as contributors to NET formation [62]. Furthermore, ROS can impair antigen-specific T cell responses by reducing the expression of the CD3ζ chains on T cells [63]. Tumor-derived factors such as IL-3, IL-6, IL-10, TGF-β, platelet-derived growth factor, and granulocyte-macrophage colony-stimulating factor (GM-CSF) promote ROS production in TANs through the STAT3 pathway [64,65,66]. ROS and peroxynitrite produced by TANs promoted tumor cell proliferation by causing nitration of T cell receptors on CD8^+^ T cells in an MC38 mouse model [67,68]. Recent evidence suggests that miRNAs released by neutrophils, such as miR-23a and miR-155, contribute to CRC progression by causing DNA double-strand breaks and inhibiting Rad51-mediated repair, while miR-155 plays a stage-specific, inhibitory role in early CRC and a promotive role in advanced stages [69,70,71].

TANs prevent CD8^+^ T cell infiltration into tumors, promote the expansion of regulatory T cells, and modulate NK cell activity, thereby contributing to an immunosuppressive tumor microenvironment. A recent study using clinical samples demonstrated that region-specific CD16^+^ neutrophils can promote CRC progression by inhibiting the cytotoxic activity of NK cells [72]. Granulocytic myeloid-derived suppressor cells (G-MDSCs), also referred to as polymorphonuclear MDSCs (PMN-MDSCs), are functionally equivalent to TANs, but represent the complexity of neutrophil heterogeneity within the TME [8]. These cells, which can be immature or mature myeloid cells, possess strong immunosuppressive properties and are found in the circulation sites, primary tumor sites, and metastatic sites [73]. In mice, G-MDSCs, characterized by the expression of CD11b^+^Gr-1^+^Ly6G^+^Ly6C^low^, are phenotypically and morphologically similar to neutrophils. In contrast, monocytic MDSCs (M-MDSCs) expressing CD11b^+^Gr-1^+^Ly6G^−^Ly6C^high^ resemble monocytes. In humans, G-MDSCs are identified by the markers HLA^−^DR^low/−^CD11b^+^CD14^−^CD15^+^, while M-MDSCs are identified by HLA^−^DR^low/−^CD11b^+^CD14^+^CD15^−^ [11]. Recently, the scRNA-seq analysis of neutrophil subsets from tumor-bearing mice identified three populations: classical neutrophils, PMN-MDSCs, and activated PMN-MDSCs [28]. The latter two exhibit potent immunosuppressive activity and are found in tumors at early stages of tumorigenesis to acquire CD14 expression, which can be a marker to distinguish them from classical neutrophils [28].

In CRC mouse models, TAN-derived extracellular vesicles also promote cancer progression, partly by increasing cancer stemness, whereas S100A9-deficient extracellular vesicles lack this effect [74]. Exosomal components, such as HSPC111 and ADAM17, amplify these effects by promoting TAN formation through reprogramming lipid metabolism and enhancing metastatic potential via E-cadherin cleavage [75,76]. Under the influence of IL-6, TANs secrete miR-93-5p-containing exosomes that induce M2 macrophage differentiation, thereby increasing the risk of CRC [77]. In advanced CRC, cancer stem cell-derived exosomes containing triphosphate RNAs prime neutrophils to promote tumor growth, which can be reversed by depleting neutrophils [78].

The role of microbiota in cancer development is increasingly being recognized, as discussed in the section on the antitumor role of TANs. Anaerobic bacteria, such as *Pseudomonas aeruginosa* and *Peptostreptococcus anaerobius*, within tumor sites can drive the recruitment of myeloid cells into the CRC microenvironment, promoting the secretion of IL-23, which in turn triggers EMT and contributes to chemotherapy resistance [79]. *Fusobacterium nucleatum* is also implicated in the early stages of inflammatory bowel disease and colorectal adenoma formation [80]. CRC patients with high levels of *F. nucleatum* in the tumor exhibit increased numbers of TANs and decreased numbers of NK cells, thereby promoting CRC progression [81,82,83].

These findings underscore the significant tumor-promoting role of TANs, which is more widely reported than their antitumor effects. Given the critical impact of TANs on tumor progression, the following sections delve into the mechanisms underlying TAN recruitment, angiogenesis, and the emerging role of NETs in cancer.

## 6. Recruitment of TANs

Recruitment of TANs to the TME is a crucial step in establishing an immunosuppressive milieu. Intriguingly, inflammation and tumors share several common mechanisms of neutrophil recruitment, and the chemokine–chemokine receptor system plays a key role in tumor progression by facilitating the accumulation of myeloid cells within the TME. Chemokines are small peptides that bind to G-protein-coupled receptors and are categorized into four subfamilies—CXC, CC, C, and CX3C—based on differences in the sequence of the first two conserved cysteines at the N-terminus [84]. The C-C motif subfamily includes chemokines such as CCL2, CCL5, and CCL15 in humans (CCL9 in mice), while the C-X-C motif subfamily includes CXCL1, CXCL2, CXCL3, CXCL5, CXCL6, CXCL7, CXCL8, and CXCL12. Various chemokine receptors, including CCR1, CXCR2, CCR2, and CXCR4, interact with their corresponding ligands and play crucial roles in immune cell trafficking and recruitment under inflammatory and cancerous conditions [40].

In particular, CXCR2 and its ligands (i.e., CXCL1, CXCL2, CXCL3, CXCL5, CXCL7, and CXCL8 in humans) play a pivotal role in the initial recruitment of TANs [17]. These Glu-Leu-Arg^+^ (ELR^+^) CXC chemokines are secreted by tumor cells and TANs, suggesting positive feedback, and are potent promoters of angiogenesis, a topic that will be discussed in the next section [85]. In mouse models of colitis-associated cancer, the elimination of neutrophil recruitment by deletion of Cxcr2 reduced overall inflammation, and thus suppressed tumors [86]. Subsequently, secretion of CXCR2 ligands by inflamed tumor cells promotes the accumulation of CXCR2^+^ TANs, which in turn inhibits the cytotoxic activity of CD8^+^ T cells and facilitates tumor progression [87]. In addition, the hypoxic response in colonic epithelial cells contributes to tumorigenesis in mouse models of colitis-associated cancer by activating HIF-2α, which further promotes neutrophil recruitment through the CXCL1−CXCR2-signaling pathway [88]. Although mouse models provide valuable insights, the biological differences should be considered when translating these results to human pathology [89].

CCR1, widely expressed on a variety of myeloid cells, is also involved in the recruitment of myeloid cells to the TME. The interaction between CCR1 and its ligands, such as CCL15 (humans) or CCL9 (mice), facilitates the recruitment of CCR1^+^ myeloid cells to primary and metastatic tumor sites. Most of CCR1^+^ myeloid cells recruited to tumors are granulocytic-MDSCs and TANs in human CRC samples [90,91,92]. In preclinical mouse models, we also demonstrated that the inhibition of CCR1-mediated myeloid cell accumulation suppressed tumor growth and metastasis [29]. Furthermore, the simultaneous inhibition of CCR1 and CXCR2 expressed on myeloid cells can result in more robust suppression of tumor growth and metastasis, highlighting the potential of combinational therapeutic strategies targeting multiple chemokine receptors [30]. In particular, targeting both CXCR2^+^ neutrophils and CCR2^+^ macrophages in pancreatic ductal adenocarcinoma improves chemotherapeutic efficacy and inhibits myeloid cell recruitment [93].

CXCR4, frequently over-expressed in malignant cells including CRC, also plays a crucial role in the regulation of neutrophil trafficking [94]. Physiologically, CXCR2-mediated neutrophil migration from the bone marrow to the peripheral blood is antagonized by CXCR4, which retains neutrophils within the bone marrow by CXCL12-expressing stromal cells [95]. Plerixafor, a small-molecule inhibitor targeting CXCR4, shows promise for mobilizing hematopoietic stem cells and could enhance the treatment of solid tumors by inhibiting CXCL12−CXCR4 signaling [96].

Genetic mutations and epigenetic alterations play a critical role in the accumulation of TANs [97]. A key tumor suppressor gene involved in the TGF-β-signaling pathway is mothers against decapentaplegic homolog 4 (SMAD4) [98]. Our group has reported that loss of SMAD4 leads to the recruitment of myeloid cells through two important chemokine pathways: the CXCL1/8−CXCR2 axis and the CCL9 (mice) or CCL15 (humans), CCR1 axis [29,31,90,91,92,99]. This promotes CRC invasion and metastasis through the recruitment of CCR1^+^ myeloid cells and CXCR2^+^ neutrophils. In both mice and humans, there is emerging evidence that Notch 3 signaling plays an important role in regulating the expression of chemokines leading to the infiltration of TANs into the TME of CRC [100]. Furthermore, transcriptional profiling reveals that epithelial NOTCH1 signaling promotes metastasis through TGF-β−dependent neutrophil mobilization by creating a TME reminiscent of poorly prognostic human CRC subtypes such as consensus molecular subtype 4 and CRC intrinsic subtype B [32]. In addition, KIAA1199, a protein involved in cell migration and tumor progression, has been identified as a key regulator of the TGF-β/SMAD3-signaling pathway. Its activation upregulates CXCL1 and CXCL3, enhances neutrophil recruitment, and promotes liver metastasis via the CXCR2 axis [101]. Similarly, another prominent genetic alteration in CRC, the Kirsten rat sarcoma viral oncogene homologue gene (KRAS) mutation, can increase the expression of CXCL3, thereby enhancing the migration of CXCR2^+^ TANs via the CXCL3−CXCR2 axis, and fostering resistance to anti-PD-1 immunotherapy in mice [33]. In KRAS-mutant CRC mouse models, both pharmacological and genetic inhibition of CXCR2 counteracts this immunosuppression and hinders tumor progression. Moreover, SLC25A22, a mitochondrial glutamate transporter, has been identified as a key player in KRAS-induced immunosuppression by driving CXCL1 transcription and is a potential therapeutic target [102]. Recent studies have discovered that stem cell markers such as doublecortin-like kinase (DCLK1) and RNA modification by methyltransferase-like 3 (METTL3) also play a role in the recruitment of CXCR2^+^ TANs to modulate tumor immunity through the CXCL1−CXCR2 axis in CRC mouse models [103,104]. These findings highlight the potential of targeting TANs and their associated pathways as a promising therapeutic strategy in the treatment of CRC.

## 7. Role of TANs in Angiogenesis and Metastasis

Angiogenesis, the formation of new blood vessels, is essential for tumor growth and metastasis. TANs also release a variety of pro-angiogenic factors, such as MMP-9, VEGF-A, and Bv8, which promote extracellular matrix degradation [24,105,106,107]. These factors contribute to metastasis by enhancing angiogenesis, protecting circulating tumor cells, reactivating dormant cancer cells, and/or helping to mobilize tumor cells to the pre-metastatic niche [73]. Clusters of neutrophils and circulating tumor cells have been observed in the circulation of breast cancer patients and mouse models, where tumor cells exhibit enhanced proliferative potential and increased efficiency in metastasis formation [108]. Recent findings have revealed that tissue-specific reprogramming of neutrophils can lead to angiogenic specialization, contributing to tumor vascularization in human CRC [109].

IL-8 (also known as CXCL8), the first-reported angiogenic chemokine, binds to its receptors CXCR1 and CXCR2, which are expressed primarily on neutrophils. CXCR2 is also expressed on endothelial cells and contributes to angiogenesis through its interaction with IL-8 [110]. In addition to angiogenesis, IL-8 is involved in cell proliferation, EMT, NET formation, immunosuppression, and metastasis via several intracellular signaling pathways [111,112]. IL-8 is considered a tumor-promoting factor and is primarily secreted by CRC cells in humans [113] but not in mice. TANs also secrete IL-8, which contributes to a positive feedback loop that amplifies neutrophil recruitment [99]. Elevated serum IL-8 levels are associated with an increased number of neutrophils within the TME and have also been identified as independent markers of reduced responsiveness to immune checkpoint inhibitors [114,115].

Cancer cells produce many factors, including IL-1β, CCL2, TGF-β, IL-6, granulocyte-colony stimulating factor (G-CSF) and GM-CSF, which affect innate immune cells, including neutrophils. Numerous studies have revealed that tumor cells or surrounding stroma secrete large amounts of G-CSF and GM-CSF, which regulates granulopoiesis by activating the myeloid transcription factor, C/EBPβ, a member of the CCAAT/enhancer binding protein (C/EBP) family [40,65,95,116]. Many of these recruited neutrophils are immature and immunosuppressive, promoting cancer metastasis [117]. In particular, integrin-β2 (CD18), a molecule expressed by neutrophils, plays a key role in cancer cell metastasis and is specifically associated with liver metastasis in a CRC mouse model [118]. G-CSF upregulates Bv8 expression in neutrophils, facilitating neutrophil recruitment to tumors and promoting angiogenesis, which ultimately result in resistance to anti-VEGF therapy in tumor xenografts [119] and spontaneous CRC mouse models [120]. The high expression of lysyl oxidase-like 4 (LOXL4) in neutrophils reveals a novel mechanism that contributes to resistance to anti-angiogenic therapy [121]. Furthermore, GM-CSF controls the overexpression of fatty acid transport protein 2 (FATP2) in neutrophils through STAT5 activation, leading to their immunosuppressive function and accelerated cancer progression in mice [122]. Further research has revealed that G-CSF signaling is modulated by IL-23 and IL-17 secreted by macrophages and T cells in the TME, thereby enhancing neutrophil recruitment and tumor progression [123].

MMP-9 is a key neutrophil protease that remodels the extracellular matrix to trigger an “angiogenic switch” and promotes tumor invasion and angiogenesis. In various murine models, TANs and TAMs are major sources of MMP-9, which is released through degranulation and binding to NETs [24]. NET-associated MMP-9 contributes to vascular dysfunction by damaging endothelial cells through cleavage and the activation of pro-MMP-2 [10] and cleaving laminin, which activates α3β1-integrin signaling and induces the proliferation of dormant cancer cells [124]. In preclinical models of CRC, neutrophil-secreted MMP-9 was also shown to activate TGF-β−mediated T cell suppression and tumor promotion, suggesting that targeting either TGF-β receptor or MMP-2/9 inhibitors could be a potential therapeutic strategy [34].

## 8. Neutrophil Extracellular Traps (NETs) and Tumor Progression

NETs are web-like filamentous extracellular structures released by neutrophils and are composed of DNA, histones, and various proteins from neutrophil granules [10]. Neutrophils decondense chromatin through a process known as NETosis, which requires neutrophil elastase activity, myeloperoxidase activity, and histone citrullination. While neutrophils serve as a defense mechanism by trapping and killing pathogens, NETs have been linked to various pathological conditions: autoimmune diseases such as rheumatoid arthritis and systemic lupus erythematosus, chronic conditions like diabetes and atherosclerosis, neurodegenerative diseases such as Alzheimer’s disease, infectious diseases like coronavirus disease 2019 (COVID-19)-related immune-thrombosis, and acute injuries including muscle damage-induced kidney dysfunction, sepsis, and acute lung injury after burns, as well as in cancer progression and metastasis. NETs are induced in cancer through a variety of inflammatory molecules, including IL-8, G-CSF, CXCL1, CXCL2, cathepsin C, and Toll-like receptor (TLR) ligands. NETs contain a variety of proteins that can directly or indirectly affect cancer cells.

NETs promote tumor growth by promoting metabolic changes within the TME. In mouse models of metastatic CRC, NET-derived neutrophil elastase activates TLR4 signaling on tumor cells, enhancing mitochondrial ATP production, and subsequently promoting primary tumor growth [125]. NET-associated neutrophil elastase stimulates the migration of human CRC cells through the activation of extracellular signal-regulated kinase (ERK) [126]. NETs interact with coiled-coil domain-containing protein 25 (CCDC25) on tumor cells and enhance cancer cell motility by activating the integrin-linked kinase (ILK)-β-parvin pathway [35]. Tandem mass spectrometry analysis of NETs isolated from the blood of healthy volunteers identified over 500 NET-associated proteins, including integrin family adhesion molecules (ITGAM, ITGB2, ITGAIIb, and ITGAL) and carcinoembryonic antigen cell adhesion molecules (CEACAM, CEACAM1, CEACAM6, and CECAM8). CEACAM1, found in NETs, directly enhances CRC cell adhesion and migration [127]. NETs can also affect angiogenesis [128], and this mechanism involves the induction of angiopoietin 1 (ANGPT1) and angiopoietin 2 (ANGPT2), potential therapeutic targets for modulating angiogenesis [129].

Furthermore, NETs contribute to the formation of an immunosuppressive niche by excluding cytotoxic CD8^+^ T cells from tumors, and act as a physical barrier that limits contact between cancer cells and immune cells, such as NK cells and T cells. Studies suggest that the inhibition of NET formation by deoxyribonuclease I (DNase I) and peptidylarginine deiminase (PAD4) can enhance the effectiveness of checkpoint inhibitors in vivo [130,131]. Additionally, tumor-associated aged neutrophils (CXCR4^+^CD62L^low^) affected by tumor-derived nicotinamide phosphoribosyltransferase (NAMPT) form two distinct types of NETs: mitochondrial-dependent vital NETs, driven by sirtuin (SIRT)1 to release mitochondrial DNA, and traditional Cit-Histone H3-dependent fatal NETs, which suggest that targeting the NAMPT–SIRT1–NET axis could be a therapeutic target [132]. NAMPT is involved in the downstream signaling of the G-CSF receptor and is essential for the tumorigenic conversion of TANs [133].

Recently, it has been reported that fibroblast growth factor 19 (FGF19) stimulates inflammatory cancer-associated fibroblasts (iCAFs), which promote neutrophil infiltration and facilitate NET formation in the liver metastatic niche. This process, driven by the release of complement proteins C5a and IL-1β, accelerates the colonization of CRC cells in the liver [134]. The interaction between NETs and dysregulated gut microbiota is also essential for the spread of CRC to the liver [135,136]. This process is facilitated by bacterial translocation from the primary tumor site, which recruits neutrophils via cytokines such as IL-1β, CCL2, TNF-α, and IL-6, thereby establishing a pre-metastatic niche in the liver [137]. Thus, NETs are gaining attention as a potential therapeutic target because of their crucial roles in tumor progression and metastasis.

## 9. Therapeutic Approach and Future Perspective

Inhibiting the polarization of neutrophils toward the N2 phenotype could shift the balance in favor of antitumor immune response. Converting a ‘cold’ tumor into a ‘hot’ tumor that responds well to immunotherapy remains a critical goal. However, a major challenge remains in selectively targeting tumor-promoting N2 TANs while sparing antitumor N1 TANs to preserve the overall immune function. To address microsatellite stable CRC resistance to the immune checkpoint blockade, combination therapies with chemokine antagonists that disrupt immunosuppressive signalings have been explored. Targeted therapies against TANs, such as CXCR2 and dual CXCR1/2 inhibitors, have successfully augmented immune checkpoint blockade therapy in both preclinical models and clinical trials [36,130] (Table 2).

CCR1-specific inhibitors include J-113863, BX471, and KM5908 [29,30,138,139,140,141]. CXCR2-specific inhibitors include AZD5069, GSK1325756 (danirixin), SB225002, SB265610, and SC656933. Dual CXCR1/2 inhibitors include DF2156A (ladarixin), SCH527123 (navarixin), reparixin (repertaxin), and SX-682 [142]. Although some of these agents, such as navarixin, SCH527123 and SX-682, were originally developed for other diseases, their application as anticancer agents, particularly targeting TANs, makes them therapeutic options for CRC therapy.

In addition to targeting TAN recruitment, other strategies have focused on inhibiting the immunosuppressive function of TANs within the TME. In a CT26 mouse model of CRC, the inhibition of phosphoinositide 3-kinases (PI3K)-δ/γ directly targeted the immunosuppressive functions of TANs, which also boosted the efficacy of anti-PD-1 therapy [143]. Novel research on melanoma has shown that inhibiting the immunosuppressive receptor CD300ld, which is specifically upregulated in TANs and activates the STAT3−S100A8/A9 axis, can improve the efficacy of anti-PD-1 therapy by remodeling the tumor immune microenvironment [144]. A clinical trial, NCT04599140, is investigating the efficacy of combined treatment with nivolumab and the CXCR1/2 antagonist, SX-682, in patients with RAS-mutated microsatellite stable CRC [145].

Furthermore, antimicrobial peptides (AMPs) have emerged as potential anticancer agents that are capable of modulating the immune microenvironment by enhancing neoantigen presentation and immune responses [146,147].

Another approach involves exploiting the plasticity of neutrophils. Mature neutrophils can be reprogrammed into multipotent progenitors by chemical stimulation, and modulation of this process offers the potential for cancer therapy [148]. For instance, the conversion of neutrophils into antigen-presenting cells through the engagement of the Fcg receptors has been shown to potentially enhance antitumor immune responses [149] Recently, chimeric antigen receptor (CAR) neutrophils have been engineered from pluripotent stem cells using CRISPR/Cas9 technology. This biomimetic CAR-neutrophil drug delivery system has been shown to be effective in glioblastoma, offering the potential for broad application in cancer therapy [150].

In summary, the inhibition of TAN recruitment, expansion, and polarization has shown promising results in preclinical and clinical settings. Advances in single-cell profiling could further refine the differentiation of TAN subtypes and improve therapeutic precision while minimizing side effects. Continued development of neutrophil-targeted therapies has great potential to improve cancer treatment outcomes.

## Figures and Tables

**Table 1:** Anti-tumor effect and pro-tumor effect of TANs.

Role	Year	Model	Mechanism	Reference
Anti- tumor	2017	Human clinical samples	Neutrophils enhance the responsiveness of CD8^+^ T cells and CD66^+^ cell infiltration in CRC is associated with increased OS	[20]
	2017	AOM/DSS-induced CAC model	CD177^+^ neutrophils suppress epithelial cell tumorigenesis in colitis-associated cancer	[26]
	2020	Human clinical samples and PDX	Anti-TGFβ attenuates tumor growth via polarization of TANs towards an anti-tumor phenotype	[27]
Pro- tumor	2021	Mouse: CT26	Neutrophils acquire immunosuppressive activity mediated by FATP2	[28]
	2020 2024	Mouse: MC38 and CMT93	TANs induce T cell suppression/Angiogenesis in the TME	[29,30]
	2014	Mouse: CMT93	Ccl9 in CRC cells recruit CCR1^+^ neutrophils which produce MMP9	[31]
	2019	Mouse: KPN model Human clinical samples	NOTCH1 signaling promotes metastasis via TGFβ-dependent neutrophil recruitment.	[32]
	2019	Mouse: iKAP, iAP models, and MC38 Human clinical samples	Oncogenic KRAS leads to high expression of CXCL3, binding CXCR2 on TANs to promote their migration	[33]
	2020	Mouse: Apc/Cdx2CreERT2 model Human clinical samples	Neutrophils suppress tumor-infiltrating T cells viamatrix metalloproteinase mediated activation of TGFβ	[34]
	2020	Human clinical samples and HCT116	NETs promote metastasis via binding CCDC25 on cancer cells	[35]
	2011	KM12L4 human metastatic CRC cells	Systemic inhibition of CXCR1/CXCR2 induced apoptosis and inhibited angiogenesis in the liver metastasis	[36]

**Table 2:** Clinical trials exploring neutrophil modulation in CRC.

Target	Agents	Mechanism	Other Interventions	Cancer Type	Phase	Identifier
CCR5	Maraviroc	Inhibitor	No (Alone)	CRC	I	NCT01736813
	Maraviroc	Inhibitor	Pembrolizumab	CRC	I	NCT03274804
	Vicriviroc (MK-7690)	Inhibitor	Pembrolizumab	CRC	II	NCT03631407
CXCR1/2	Navarixin	Inhibitor	Pembrolizumab	Various (including CRC)	II	NCT03473925
	SX-682	Inhibitor	Alone or Nivolumab	CRC	I/II	NCT04599140
CXCR4	Plerixafor	Inhibitor	No (Alone)	Various (including CRC)	I	NCT02179970
IL-1R	Anakinra	Inhibitor	LV5FU2 and Bevacizumab	CRC	II	NCT02090101
STAT3	Napabucasin (BBI-608)	Inhibitor	Chemotherapy (FOLFIRI)	CRC	III	NCT02753127
	Danvatirsen (AZD9150)	Inhibitor	No (Alone)	Various (including CRC)	I/II	NCT01563302
	Danvatirsen	Inhibitor	Alone or Durvalumab	Various (including CRC)	I	NCT03394144
IDO1	Epacadostat	Inhibitor	Pembrolizumab and chemotherapy	Various (including CRC)	I/II	NCT03085914
ARG1	INCB001158	Inhibitor	Pembrolizumab	Various (including CRC)	I	NCT02903914
	ARG1 vaccine	Peptide vaccine	Adjuvant Montanide ISA-51	Various (including CRC)	I	NCT03689192
TRAIL receptor 2	DS-8273a	Agonist	No (Alone)	Various (including CRC)	I	NCT02076451
(DR5)	DS-8273a	Agonist	Nivolmab	CRC	I	NCT02991196
	CS-1008	Agonist	No (Alone)	CRC	I	NCT01220999
